# Timing of diffusion tensor imaging in the acute spinal cord injury of rats

**DOI:** 10.1038/srep12639

**Published:** 2015-07-29

**Authors:** Xiao-Hui Li, Jian-Bin Li, Xi-Jing He, Fang Wang, Sheng-Li Huang, Zhi-Lan Bai

**Affiliations:** 1 Departments of Radiology, the Second Affiliated Hospital, School of Medicine, Xi’an Jiaotong University, Xi’an 710004, China; 2 Department of Orthopaedics, Luochuan County Hospital, Luochuan County 727400, China; 3 Department of Orthopaedics, the Second Affiliated Hospital, School of Medicine, Xi’an Jiaotong University, Xi’an 710004, China

## Abstract

The aim of this study was to evaluate the characteristics of magnetic resonance diffusion tensor imaging (DTI) in acute spinal cord following a thoracic spinal cord injury (SCI), and to determine the optimal time of examination. Sprague-Dawley rats were used as experimental animals and contusion injuries were made at the T10 vertebral level. The rats were divided into control, mild injury, moderate injury, and severe injury groups. Spinal magnetic resonance DTI was scheduled at 6, 24 and 72 hours (h) post-SCI, and the DTI parameters such as fractional anisotropy (FA) and apparent diffusion coefficient (ADC) were calculated, and the diffusion tensor tractography (DTT) of the spinal cord was also generated. We observed a significant decrease of FA in all the three injured groups, and the FA at 24 h post-SCI exhibited the greatest decrease among different set times. For ADC, only the group of severely injured rats saw a significant decrease at 24 and 72 h compared with the control group. DTT showed interruption of nerve fiber tracking in the injured groups. This study demonstrates that FA can differentiate various grades of SCI in the early stage, and 24 h after injury might be the optimal time for identifying injury severity.

Imaging is a critical component of clinical evaluation on spinal cord injury (SCI). Magnetic resonance imaging (MRI) has been widely employed as an ideal noninvasive technique for examining SCI. Although conventional MRI plays an essential role in the diagnosis and treatment of SCI, it is not effective or reliable in accurately assessing the functional integrity of the injured spinal cord in the early stage after SCI due to the temporary flaccid paralysis that accompanies spinal shock. In the acute stage, predicting the severity of injury is very important to the clinical management of patients because a reliable indication of the extent of injury will facilitate timely decision and application of appropriate treatment modalities. To effectively assess the severity of SCI, sensitive methods are needed to reveal changes in the neurological structure. With the technical advancement in the field of radiology, diffusion tensor imaging (DTI) has been developed and used as an accurate, non-invasive measuring approach in SCI.

DTI is an imaging method based on the diffusion of water molecules in tissues. It quantifies the diffusion of water molecules in both the longitudinal and transverse directions to the axis of neuronal axons. Frequently used quantitative DTI parameters include fractional anisotropy (FA) and apparent diffusion coefficient (ADC). FA reflects the anisotropy of the diffusion, and ADC reflects the magnitude of the diffusion. Ever since DTI was described by Basser in 1994^1^, quantitative DTI parameters have been utilized extensively in the research of brain development and diseases^2^,^3^,^4^,^5^,^6^, as well as nerve trauma^7^,^8^. Studies about DTI in SCI are comparatively fewer. Some experimental studies have reported that DTI is more sensitive than conventional MRI in detecting SCI^9^,^10^,^11^ and predicting the severity of injury^12^. However, none of the studies have paid attention to the timing of DTI examination in the acute stage of SCI.

The present study was aimed to look into the importance of timing of DTI in SCI. We performed experimental thoracic SCI contusion of varying severities in rats, followed by DTI evaluation at the injury epicenter. Quantitative DTI parameters including FA and ADC were calculated, and diffusion tensor tractography (DTT) in the injury site was reconstructed. Timing of examination was investigated by observing the differences of FA values at different time points, and the optimal time of examination was then identified.

## Results

### Conventional MRI

**Figure 1:**
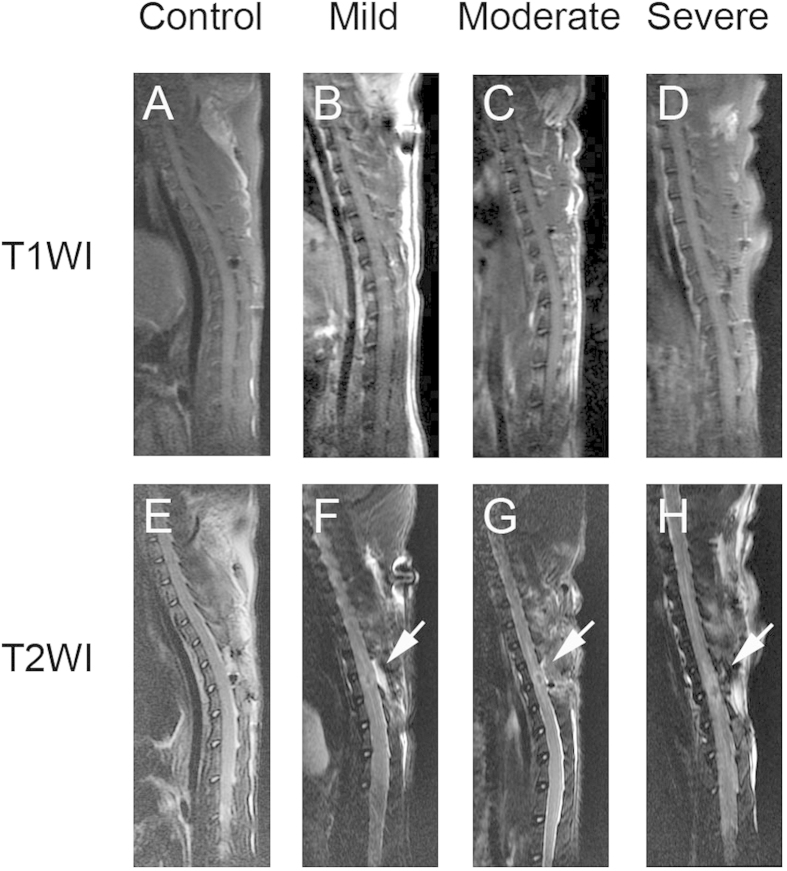
Conventional magnetic resonance imagings from all the groups at 6 h after injury. The T1-weighted images (T1WI) depicted less noticeable changes of the signal intensity in SCI (**B**–**D**). In T2-weighted images (T2WI) of SCI, injured groups showed a hypointense region within the central cord parenchyma, surrounding a hyperintense region corresponding to the site of contusion (**F**–**H**).

Rats in the control group presented normal spinal cord morphology. There was no evidence of intramedullary pathology. T1WI at 6 hours (h) post-surgery showed no clear differentiation between uninjured and injured regions (Fig. 1). In contrast, conventional midline sagittal T2WI in the injured groups demonstrated focal decreased signal of the spinal cord with surrounding abnormal increased signal intensity corresponding to the site of contusion. Circumambient hyperintense regions extended in both rostral and caudal directions from the injury epicenter in the severely injured group.

### DTI

The mean FA of the uninjured spinal cords in the control group was 0.66 ± 0.04. FA in the three SCI groups (Table 1) exhibited significant reduction at 6, 24, and 72 h after injury compared with that in the normal control (*P* < 0.05), and the values in the severely injured group was significantly lower than that in the moderately injured and mildly injured groups (*P* < 0.05). The decrease of FA at different time points in the injured groups was consistent with the visual inspection of fiber tracking. In other words, FA values altered significantly in the injured groups; the more severe the injury was, the more the FA values decreased. As for the different time points, an initial decrease in FA was observed at 6 h post injury, and the maximum reduction was seen at 24 h. The difference between the two time points was significant (*P* < 0.05). However, there was no significant difference between the FA values acquired at 24 h and 72 h.

The mean value of ADC in the control group was 1.04 ± 0.06. At the set time points, ADC did not show significant differences (*P* > 0.05) among the control, mildly injured, and moderately injured groups (Table 1). However, significant decreases were observed at 24 h and 72 h in the severely injured group (P < 0.05).

### DTT

**Figure 2:**
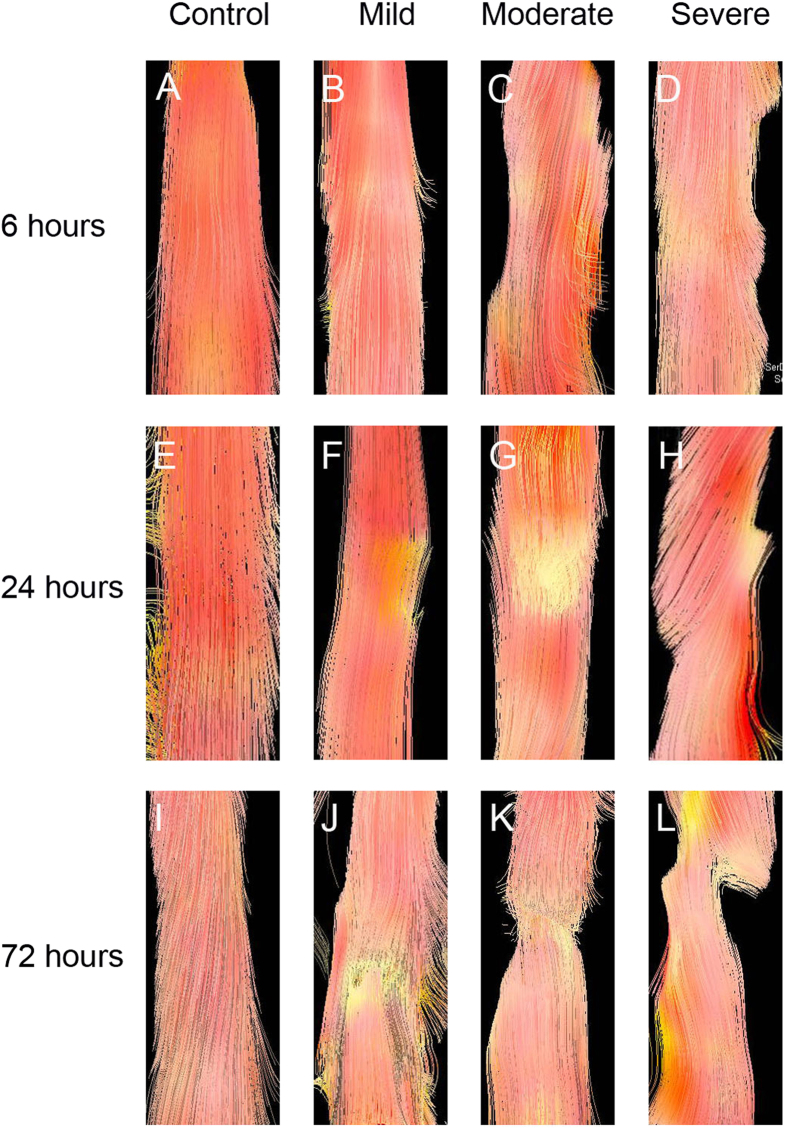
Diffusion tensor tractography of SCI in the white matter tracts. Diffusion tensor tractography shows the integrity change of white matter fiber tracking in SCI. The white matter tracts are observable and appeared disconnected (**F**–**H**), (**J**–**L**).

DTT of the spinal cord enabled us to visualize the fiber tracking of spinal cord tracts (Fig. 2). The control rats presented well-organized fiber tracking of the spinal cord tracts, while the injured rats with different severities of injury showed different characteristics at different time points. At 6 h after injury, DTT of the mildly injured rats demonstrated uninterrupted fiber tracking of nerve tracts, but that in the zones of moderate and severe injuries showed irregularity in nerve fiber tracks. At 24 h, DTT clearly demonstrated the lack of continuity of nerve fiber tracking, and the tractography at 72 h revealed that white matter fiber tracking were disrupted at the injury site. Especially, at the site of severe injury, DTT showed abrupt cessation of fiber tracking at 72 h after injury.

## Discussion

Conventional MRI has been used as a major approach in the diagnosis and treatment of SCI. However, it may not be effective or reliable in evaluating the axonal disruption in the early stage after SCI. The recently developed DTI method can reflect the subtle pathological and physiological changes in the injured spinal cord. It is considered the ideal imaging tool to assessing the severity of SCI. In the present study, we examined the sequential FA and ADC values, two DTI parameters, at the lesion epicenter of SCI rats, and found that there were different patterns of changes in these parameters in terms of different injury severities and time points. Our DTT results indicated the presence of interruption of local fibers in the SCI rats. The decreased FA value was also consistent with the formation of interruption of local fibers. In addition, our study indicated that quantitative FA was a sensitive and specific biomarker for SCI. The DTI results suggest that significant injured fiber occurs at 24 h after SCI. To the best of our knowledge, this is the first study performed on determining the optimal time of DTI examination in acute SCI.

We used contusion injury model in this study because this model compared with other SCI models, more closely resembles the histopathology and imaging findings of human traumatic SCI more closely^13^. The model was prepared by inducing a contusion injury at the thoracic (T10) level in adult male rats. During the surgical process, the rats were maintained under general anesthesia and placed at supine position to minimize the intervention of bulk motion, such as cardiac and respiratory motion^9^,^14^. As we all know, SCI is a dynamic injury in which tissue pathology continues changing months after the initial trauma; the process is complex, including pathology such as demyelination, axonal damage, hemorrhage, necrosis, astrogliosis, etc^15^. Based on the aim of this study, we restricted our DTI measurements to the lesion site, and DTI was only examined within 72 hours after injury. Image acquisition of SCI was carried out without analysis of corresponding histological sections. Histology is not an optimal choice in experimental SCI as large numbers of animals must be sacrificed at each experimental time point^16^. Fortunately, DTI makes continuous monitoring of SCI in a specific animal possible, significantly contributing to the study of SCI.

Conventional MRI is considered the gold standard imaging modality in the assessment of SCI^17^, yet it has not been shown consistently relate to the clinical findings post SCI^18^. T2WI is known to detect hemorrhage and edema in the acute stage^19^. In our study, significant signal abnormalities were observed only in the rats with moderate and severe injuries. T2WI sequences revealed hypointense regions at the injury epicenter with surrounding hyperintense indicative of central syrinx formation and preserved white matter fibers, respectively. However, the presence of spinal cord T2 hyperintensity does not predict functional outcomes^12^. Although conventional MRI can depict several histological changes of the spinal cord^20^,^21^,^22^,^23^,^24^,^25^,^26^,^27^, the data will be mostly qualitative. But DTI provides quantitative information about tissue microstructure, such as axons. DTI is an imaging method based on the diffusion of water molecules in tissues. It has been demonstrated to be a more sensitive imaging modality for SCI than standard T2WI for SCI. As reported in our prior study, DTI can assess spinal cord integrity and monitor microstructural changes^28^. The injury in the spinal cord can be quantified through the value of FA, a DTI parameter, in living animals. In addition, DTI can be performed in the process when a patient receives a conventional MRI. It requires only a few more minutes. DTI provides a powerful tool to examine the injured areas in the acute stage of SCI using molecular techniques.

In the present study, we examined the sequential FA and ADC values at the lesion epicenter. The changes of the two DTI parameters presented different patterns. ADC might not always reflect severity in the acute stage, and FA may make it possible to quantify the severity of SCI. FA reflects white matter integrity, including axonal structure damage and demyelination. Reduced FA is indicative of nerve degeneration and axonal loss^29^. In our study, the FA values decreased with the increasing severity of injury. The lowered FA values are due to the breach in the longitudinal tracks. Comparatively, DTI is more sensitive than conventional MRI in detecting SCI. In the mild injury group, the FA values decreased significantly but MRI demonstrated no evidence of signal abnormalities. We have also found that FA and ADC do not always change in parallel. ADC did not show any significant changes with time in both the mild and moderate injury groups, suggesting that ADC was not effective in assessing the injuries. In all the SCI groups, FA decreased significantly at 6 h post injury even though no changes in ADC were observed, suggesting that FA value is a sensitive marker of SCI and FA is more strongly related to the severity of injury. Our observation is in agreement with previous results^30^. We thus recommend the use of DTI-derived FA, not ADC, as a biomarker of SCI.

While the utility of DTI has been demonstrated in various studies, the optimal time of examination has not been reported. In the present study, we determined the optimal timing of examination based on the sensitivity of DTI parameters to the pathological changes in SCI. Since FA presented a better ability than ADC in detecting SCI, we determined the optimal time of DTI based on FA values. Our results showed that the FA values were reduced more significantly at 24 h than at 6 h in all the injury groups, indicating that FA values at 24 h after injury can accurately reflect the severity of cord injury. Therefore, we suggest 24 h after injury as the optimal time for evaluating the axonal disruption.

The use of DTI with the corresponding tractography provides additional information that conventional T2 sequences cannot offer. Routine MRI does not provide direct measurement of axonal integrity^31^, while DTT gives insights into tissue structure at a microscopic scale, which can clearly demonstrate the specific injury area. DTT can actually differentiate interrupted nerve fiber tracking from intact regions. The detection of distortion and interruption of the injured spinal cord by DTT matches the findings by T2WI. Although DTT does not reflect the actual number of spared axons fibers after SCI, it allows analyzing the overall shape of the spinal cord around the contusion site. Therefore, DTT could present the degree of white matter damage following graded contusive SCI.

This study had the following limitations. First, no histological examination was performed. Second, this study is an initial step toward establishing the optimal time for DTI examination to evaluate the extent of SCI in the acute stage. Further studies in human beings are required to know the efficacy, and reproducibility of these results.

In conclusion, DTI may have significant advantages over the conventional MRI in assessing the severity of injury in the acute stage of SCI. DTI can differentiate between mild, moderate, and severe types of injury to the spinal cord, and it is a sensitive measure of SCI in the acute stage. Among the three time points set in this study, 24 h post injury appears to be the optimal time for examination. Our results are very encouraging and warrant further work with a large cohort.

## Materials and Methods

### Animal grouping

This study was approved by the Animal Experiment Committee of Xi’an Jiaotong University. Adult male Sprague-Dawley rats weighing between 250 and 300 g were used. Principles of laboratory animal care were followed and all procedures were conducted in conformity with the guidelines established by the National Institutes of Health, and every effort was made to minimize animal suffering. Twenty-four rats were randomly divided into four groups with six rats in each group. The control group received laminectomy with spinal cord uninjured. The three SCI groups were set as mildly injured, moderately injured, and severely injured, in which spinal cord injury was caused by a weight (10 g) dropping from the height of 12.5, 25, and 50 mm respectively, with SCI impactor (NYU weight-drop device, New York University, New York, USA).

### Experimental treatment

The NYU weight-drop SCI modeling (10 g rod dropped from a pre-determined height) was used to create contusions corresponding to mild, moderate, and severe spinal cord injuries. All surgical procedures were performed under aseptic conditions. The rat was anesthetized with intraperitoneal injection of chloral hydrate (100 g/L) at 300 mg/kg body weight and was placed in prone position. After shaving and routine aseptic preparation, a standard dorsal midline approach was performed to expose the spinous process and lamina from the eighth thoracic vertebra (T8) to the first lumbar vertebra (L1). The dorsal spinous processes of T9 through T11 were removed with rongeurs. A dorsal laminectomy was performed to expose the spinal cord and overlying dura, and the dura must not be injured. A spinal contusion was created at T10 segment with impactor. Hemorrhage and edema in the injured area, as well as the emergence of wagging tail reflection and lower limbs such as flutter, indicated the success of modeling. The incision was then closed in layers. After surgery, an antibiotic agent (Cefazolin) was administered daily by intramuscular injection. Rats had free access to food and water, and underwent manual bladder evacuation twice every day. Animals were given adequate analgesia postoperation. All the rats could not be assessed by clinical scoring at the time points of imaging, because of spinal shock in the acute phase of the disease.

For rats in the control group, laminectomy was done but the spinal cords were uninjured.

### MRI and DTI scan

Conventional MRI and DTI scans were conducted *in vivo* at 6, 24, 72 h after the surgical treatment. Animals were mounted in supine position within the scanner. All MRI examinations were performed on a 3.0 Tesla MR Scanner (Signa, GE Medical Systems, Milwaukee, Wisconsin, USA) followed in configuring dedicated scanner at 3 Tesla for studying rat models. Identical anatomical markers for tracing the lesion, which were clearly visible on MRI, were used for all imaging sessions to obtain anatomically matched images at each time point.

Conventional MRI scan, including T1-weighted and T2-weighted images (T1WI and T2WI) was completed with the SE sequence. Sagittal T2WI was acquired using the following parameters: TR/TE of 2200 ms/126 ms, image matrix of 256 × 256, and 6 contiguous slices, with a slice thickness of 1.5 mm. The parameters for sagittal T1WI were: TR/TE of 440 ms/11.1 ms, image matrix of 256 × 256, and 6 contiguous slices with a slice thickness of 1.5 mm.

DTI images were acquired with identical geometry as the anatomical images using single shot spin-echo planar imaging (EPI) sequence with TR/TE of 4000 ms/88 ms, slice thickness of 3 mm, b factor of 1000 s/mm^2^, bandwidth of 200 kHz, 25 gradient encoding directions, acquisition matrix of 64 × 64, and field of view 10 mm × 10 mm.

### DTI analysis

After image acquisition, the data were transferred to an independent workstation to calculate the DTI indices. DTT of the spinal cord was generated using the FACT algorithm implemented in Volume-One software, and FA threshold <0.2 and stopping angle of >25° were used as parameters.

### Statistical analysis

Data were expressed as mean ± SD, and were analyzed using SPSS 16.0. Wilcoxon Signed-Rank test was performed to evaluate the difference of FA and ADC values between the injured and control groups or different time points within each injured group. Kruskal-Wallis test was used to compare data among the three injured groups. P < 0.05 was considered as statistically significant.

## Additional Information

**How to cite this article**: Li, X.-H. *et al*. Timing of diffusion tensor imaging in the acute spinal cord injury of rats. *Sci. Rep*. **5**, 12639; doi: 10.1038/srep12639 (2015).

## Figures and Tables

**Table 1:** Fractional anisotropy and apparent diffusion coefficient values in the all injury groups.

Group	Fractional anisotropy			Apparent diffusion coefficient		
	6 hours	24 hours	72 hours	6 hours	24 hours	72 hours
Mild	0.55 ± 0.03	0.47 ± 0.03	0.46 ± 0.02	1.12 ± 0.14	1.06 ± 0.08	1.08 ± 0.23
Moderate	0.45 ± 0.05	0.39 ± 0.02	0.39 ± 0.03	0.95 ± 0.25	0.95 ± 0.18	0.98 ± 0.19
Severe	0.38 ± 0.03	0.32 ± 0.03	0.31 ± 0.02	0.85 ± 0.19	0.70 ± 0.14	0.76 ± 0.14
